# An ionising radiation-induced specific transcriptional signature of inflammation-associated genes in whole blood from radiotherapy patients: a pilot study

**DOI:** 10.1186/s13014-021-01807-4

**Published:** 2021-05-03

**Authors:** Lourdes Cruz-Garcia, Christophe Badie, Selvakumar Anbalagan, Jayne Moquet, Lone Gothard, Grainne O’Brien, Navita Somaiah, Elizabeth A. Ainsbury

**Affiliations:** 1 PHE CRCE, Chilton, Didcot, Oxford, OX11 0RQ UK; 2 Institute of Cancer Research and The Royal Marsden NHS Foundation Trust, Sutton, London, SM2 5NG UK; 3 Environmental Research Group within the School of Public Health, Faculty of Medicine at Imperial College of Science, Technology and Medicine, London, UK

**Keywords:** Ionising radiation, Blood, Gene expression, Transcription, NCounter, RT-qPCR, Inflammation, Radiotherapy, Cancer

## Abstract

**Background:**

This communication reports the identification of a new panel of transcriptional changes in inflammation-associated genes observed in response to ionising radiation received by radiotherapy patients.

**Methods:**

Peripheral blood samples were taken with ethical approval and informed consent from a total of 20 patients undergoing external beam radiotherapy for breast, lung, gastrointestinal or genitourinary tumours. Nanostring nCounter analysis of transcriptional changes was carried out in samples prior and 24 h post-delivery of the 1st radiotherapy fraction, just prior to the 5th or 6th fraction, and just before the last fraction.

**Results:**

Statistical analysis with BRB-ArrayTools, GLM MANOVA and nSolver, revealed a radiation responsive panel of genes which varied by patient group (type of cancer) and with time since exposure (as an analogue for dose received), which may be useful as a biomarker of radiation response.

**Conclusion:**

Further validation in a wider group of patients is ongoing, together with work towards a full understanding of patient specific responses in support of personalised approaches to radiation medicine.

## Introduction

A variety of different biological and physical retrospective tools are available to assess individual radiation doses following a radiation accident or incident [1–4]. In recent years, transcriptional changes in blood have been identified as a promising biomarker for radiation response to support biodosimetric assessment of individual doses in accidental exposure scenarios [5, 6]. The responsiveness of FDXR to ionising radiation at the transcriptional level in human blood was recently reported to provide accurate in vivo dose estimates and providing the first in vivo dose response in humans [7]. Transcript variants of this gene have also shown a remarkable potential as standalone biomarkers for ionizing radiation exposure screenings [8]. The influence of several potential confounding factors (cancer condition, sex, simulated bacterial infection (lipopolysaccharide), and curcumin, an anti-antioxidant agent) on radiation dose estimation using in vivo validated transcriptional biomarkers was also investigated, with the outcome that such confounding factors should not prevent the use of transcriptional responses for emergency triage purposes [9]. Most recently, a new protocol for rapid gene expression-based dose estimation in human blood was reported [10], together with the generation of a transcriptional radiation exposure signature in human blood using long-read nanopore sequencing including several new genes representing ideal biomarkers of radiation exposure [11].

Although radiation-responsive genes such as FDXR have been reported in human white blood cells in radiotherapy patients, they have limited inter-individual variability in response; apart from early responsive genes such as CDKN1A which may be help to predict the severity of acute skin radiation toxicity [12], they are not informative of inter-individual variability in normal tissue sensitivity to radiation exposure. We previously reported that several genes associated with inflammatory processes (ARG1, BCL2L1, and MYC) present a long-term modification of transcriptional expression [13] towards the end of the radiotherapy treatment. In addition, there is strong evidence of radiation-induced inflammation feeding into innate and adaptive antigen-specific immune responses [14–18].

In this communication, we present a panel of inflammation-associated genes which are radiation responsive after exposure in vivo in radiotherapy patients, and their response is independent of cancer type but dependent on time post-exposure.

## Methods

The general conditions for ethical approval, patient selection and informed consent, blood sampling and individual patient dosimetry were described in detail in Moquet et al. [19]. Briefly, blood samples from five breast, four endometrial, five lung, three prostate, two oesophagus and one colon cancer patients, treated with Intensity Modulated Radiotherapy (IMRT) using a linear accelerator (LINAC) were collected in PaxGene tubes according to the manufacturers’ protocol (Qiagen, PreAnalytiX GmbH, Hilden, Germany) at four different time points during the course of the treatment: before the start of the treatment, 24 h after the first fraction, just before the fifth or sixth fraction and the last fraction. One blood sample per patient was taken at each time point. The prescribed doses for each patient are described in Table 1. Patients did not receive previous radio- and/or chemotherapy treatments except for one of the lung cancer patients who received chemotherapy five weeks before the start of radiotherapy. Blood was collected at the Royal Marsden Hospital and Institute of Cancer Research (Surrey, UK) with written informed consent from all subjects as part of the RTGene study (ClinicalTrials.gov NCT02780375), which was ethically approved by the South Central-Hampshire B Research Ethics Committee (16/SC/0307).

**Table 1:** Blood collection sampling times and prescribed doses, doses per fraction and number of doses for breast, lung, endometrial, prostate, oesophagus and colon cancer patients also described in Moquet et al. [19]

Cancer type	Number of patients	Radiotherapy scheme	Fractions	Total dose, Gy	Blood collection sampling times			
					Before	24 h after	Before 5–6 fraction	Before last
Breast (one sided)^a^	4	IMRT	15	40	Before first fraction	24 h after first fraction 2.67 Gy	Before 5–6 fractions (10.68–13.35 Gy)	Before last fraction (37.38 Gy)
Breast (bilateral)^b^	1	IMRT	15 × 2	40 × 2	Before first fraction	24 h after first fraction 2.67 Gy × 2	Before 5–6 fractions (10.68–13.35 Gy) × 2	Before last fraction (37.38 Gy) × 2
Lung	5	IMRT	20	55	Before first fraction	24 h after first fraction 2.75 Gy	Before 5–6 fractions (11–13.75 Gy)	Before last fraction (52.25 Gy)
Endometrium	4	IMRT	25	45	Before first fraction	24 h after first fraction 1.8 Gy	Before 5–6 fractions (7.2–9 Gy)	Before last fraction (43.2 Gy)
Prostate	3	IMRT	20	60	Before first fraction	24 h after first fraction 3 Gy	Before 5–6 fractions (12–15 Gy)	Before last fraction (57 Gy)
Oesophagus^c^	2	IMRT	12/5	36/20	Before first fraction	24 h after first fraction 4/3 Gy	Before 5–6 fractions (16–20/16 Gy)	Before last fraction (44 Gy)
Colon^d^	1	IMRT	15	40	Before first fraction	24 h after first fraction 2.67 Gy	Before 5–6 fractions (10.68–13.35 Gy)	Before last fraction (37.38 Gy)

^a^For one breast cancer patient, the third sample was taken 70 min post 6th fraction

^b^The patient with bilateral breasts irradiated, she received 40 Gy in 15 fractions to each breast at the same time

^c^One of the oesophagus cancer patients was only prescribed 5 fractions, and their 4th and final samples was therefore collected just before the 5th fraction

^d^Last fraction sample was collected three days before the last fraction

### RNA isolation and reverse transcription

Total RNA was extracted with the PAXgene Blood miRNA kit (Qiagen, PreAnalytiX GmbH, Hilden, Germany) using a robotic workstation Qiacube (Qiagen, Manchester, UK). The quantity of isolated RNA was determined by spectrophotometry with a ND-1000 NanoDrop and quality was assessed using a Tapestation 220 (Agilent Technologies, CA, USA). cDNA was prepared from 350 ng of the total RNA using High Capacity cDNA reverse transcription kit (Applied Biosystems, FosterCity, CA, USA) according to the manufacturer’s protocol.

### nCounter analysis

Samples were analysed by the nCounter Analysis System (NanoString Technologies^®^, Inc., Seattle, WA, USA) according to the manufacturers’ guidelines. The samples were run using 100 ng RNA per sample on the Human Inflammation V2 panel, which consists of 249 genes.

### Quantitative real-time polymerase chain reaction

SYBRGreen RT-qPCR was performed using Rotor-Gene Q (Qiagen, Hilden, Germany). All reactions were run in triplicate using PerfeCTa SYBR^®^ Green SuperMix (Quanta Biosciences, Inc., Gaithersburg, MD, USA) with primer sets for target genes at 500 nM concentration each. Cycling parameters were 2 min at 95 °C, then 40 cycles of 10 s at 95 °C and 60 s at 60 °C. Data were collected and analysed by Rotor-Gene Q Series software. Fold of change values were calculated using the delta–delta Ct method [20]. The primer sequences for SYBRGreen analysis were HPRT1 F: 5′ TCAGGCAGTATAATCCAAAGATGGT 3′, R: 5′ AGTCTGGCTTATATCCAACACTTCG 3′; IL7 5′ CTCCCCTGATCCTTGTTCTG 3′, R: 5′ TCATTATTCAGGCAATTGCTACC 3′; CD40LG F: 5′ CACCCCCTGTTAACTGCCTA 3′; R: 3′ CTGGATGTCTGCATCAGTGG 5’.

### BRB-ArrayTools and MANOVA

Statistical analysis was performed with BRB-ArrayTools [21], using the class comparison function multivariate permutation tests with a false discovery rate (FDR) < 0.05 and two-way mixed model Analysis of Variance (ANOVA) to identify genes for which there were statistically significant changes in gene expression (up or down regulation; *p* < 0.05) associated with number of radiotherapy (RT) fractions and time since exposure [22]. General Linear Model Multivariate Analysis of Variance (GLM ANOVA) and Multivariate Analysis of Variance (MANOVA) was then carried out with Minitab18^®^, to identify panels of genes significantly associated (*p* < 0.05) with radiation exposure, taking into account radiotherapy patient group by type of cancer treated (breast, lung, gastrointestinal or genitourinary tumours) and time since exposure (just before exposure, 24 h post-exposure, and just before the final fraction -range 3–5 weeks for all patients).

### Differential expression and pathway analysis

Nanostring nCounter nSolver 4.0 (Nanostring Technologies) with the Advanced analysis pluggin (version 2.0.134) was used to perform the differential expression (DE) and pathway analysis. DE analysis includes several multivariate linear regression models to identify significant genes (mixture negative binomial, simplified negative binomial, or log-linear model). FDR *p*-value adjustment was performed with Benjamini–Yekutieli method [23]. Statistically significant, differentially expressed genes were defined as those with expression levels corresponding to a log2 ratio > 0.5 or <  − 0.5 and *p*-value < 0.05.

Gene set analysis (GSA) is a quantitative summary of DE for gene sets. Gene set’s global significance score for a covariate is calculated as the square root of the mean squared t-statistic of genes. Global and directed significance scores were calculated for each pathway. Pathway scores were used to summarize data from a pathway's genes into a single score. Pathway scores were calculated as the first principal component of the pathway genes' normalized expression and standardized by Z scaling. Pathway scoring helps to see how pathway scores change across samples. Increasing score corresponds to mostly increasing expression.

### Immune cell type profiling

The cell type profiling module in Nanostring nCounter nSolver 4.0 advanced analysis was used to quantify cell populations using marker genes. Raw cell type measurements are calculated as the log2 expression of each cell type’s marker genes and show the estimated abundances of each individual cell type between samples.

## Results

### BRB-ArrayTools and MANOVA

A total of 29 genes were identified by BRB-ArrayTools as being significantly down or up regulated in response to ionising radiation exposure, with FDR < 0.05. GLM ANOVA then revealed a subset of down-regulated genes only for which both time since exposure and type of cancer were statistically significant, which indicates that these genes may be informative in understanding patient group specific responses. MANOVA on these genes reveals that this combined set of 7 genes (MYC, CD40LG, CCL4, IL7, TCF4, CCR7 and FASLG) is together statistically significantly reliant on both time post-exposure (*p* = 0.042) and cancer type (*p* < 0.001). For the up-regulated genes, no significant effects were identified for time post-exposure.

### Differential gene expression analysis

**Fig. 1:**
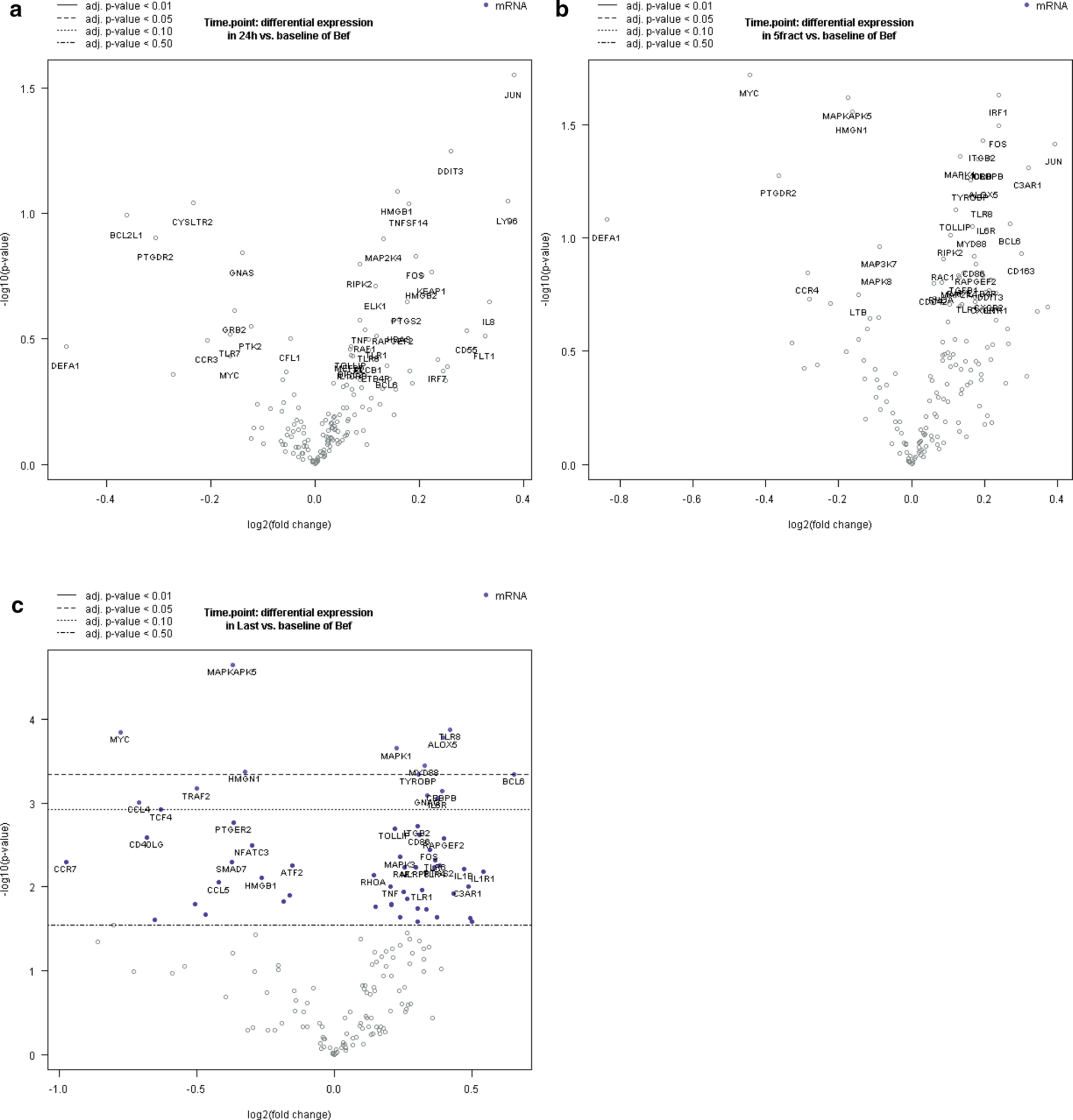
Volcano plots displaying − log10 of the *p*-value and log2 of the FC of the differential expression analysis between the time points “before the start of the treatment” and **a** 24 h after first fraction, **b** before 5th–6th faction, **c** before last fraction. Horizontal lines indicate various adjusted *p*-value thresholds when there are significant differences. The 40 most statistically significant genes are labelled in the plot (blue dots) (**c**)

DE analyses revealed a set of genes differentially expressed in blood samples at the time point before the last fraction (Fig. 1a–c). The genes TLR8, ALOX5, TYROBP, MAPK1, MYD8B, BCL6, HHGNI, MYC and MAPKAPK5 presented an up- or down-regulation (*p*-value < 0.05) independently of the cancer type (Fig. 1c) at the last samples time point. The previous time points, 24 h and before 5th–6th fractions (Fig. 1a, b) didn’t present any significantly modulated genes, probably due to the lower number of fractions received to induce an inflammatory response detectable in a blood sample. However, only MYC and BCL6 showed a fold change regulation above 1log2 FC > 0.5 or <  − 0.5.

### Gene set analysis (GSA)

**Fig. 2:**
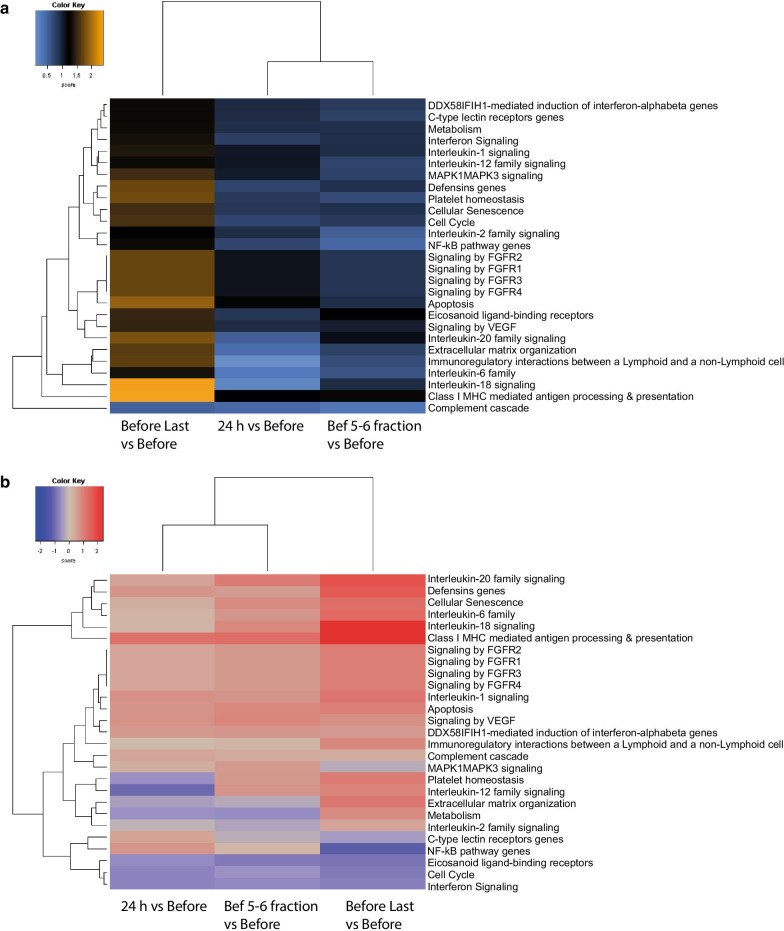
GSA: Gene set analysis with global significance scores and directed global significance scores. **a** Global significance score plot: orange denotes gene sets whose genes exhibit extensive differential expression with the covariate, blue denotes gene sets with less differential expression. **b** Directed global significance score plot: red denotes gene sets whose genes exhibit extensive over-expression with the covariate, blue denotes gene sets with extensive under-expression

Differentially expressed gene sets were observed only when comparing the before time point with the last time point (before last fraction) (Fig. 2a). These results highlighted the Interleukin 18 family and Class I MHC mediated antigen processing and presentation genes, apoptosis, interleukin 20 family, platelet homeostasis and defensins pathways with the highest scores (Fig. 2a). Directed global significance scores indicated that these pathways are upregulated at the last time point (Fig. 2b).

### Pathway scoring

**Fig. 3:**
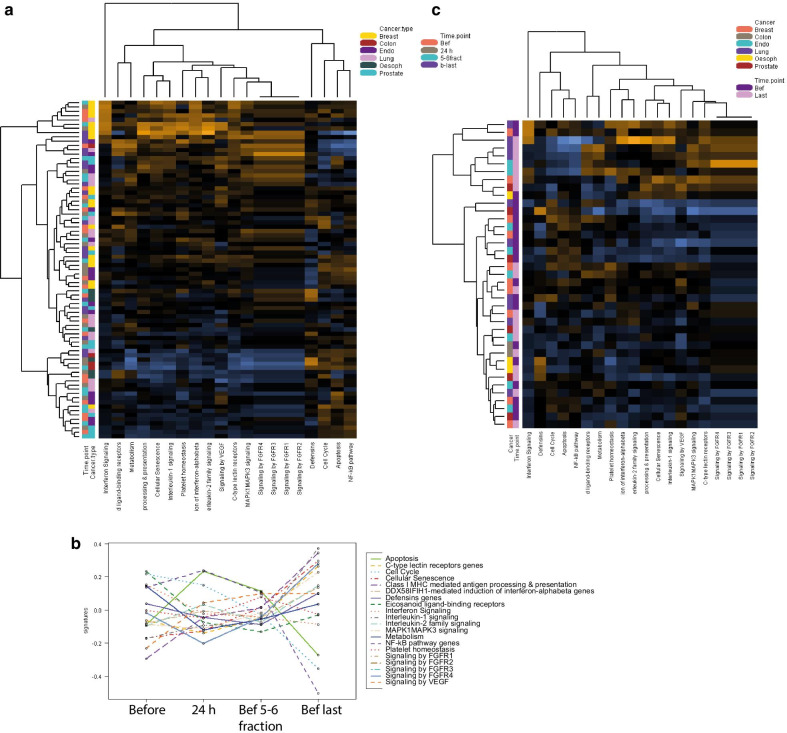
Pathway changes in the different time points **a** Heatmap of pathways scores including the 4 time points and the 6 cancer types: orange indicates high scores; blue indicates low scores. Scores are displayed on the same scale via a Z-transformation. **b** Individual pathway scores for each time point **c** Heatmap of pathways scores comparing the time point “before” and “before last fraction” together with the 6 cancer types

Pathway score clustering showed a general separation of the time points for some of the cancer types, indicating higher scores at the last time point and also for breast cancer patients (Fig. 3a). When we compared pathway scores to time points (Fig. 3b) we observed a decrease in NF-kB, cell cycle and apoptosis at the last time point. However, the scores in interleukin 1 signalling, Class I MHC mediated antigen processing and presentation genes, cellular senescence, signalling by FGFR4, C-type lectin receptors and MAPK1MAPK3 signalling pathways were increased in the last time point (Fig. 3b).

Pathway scoring between only the first and last time points (Fig. 3c) showed two clusters one of higher scores in the last time points for a group of samples and a low score cluster for the first time point (before time point) which shows a switch on of several inflammation related pathways at the end of the radiotherapy treatment.

### Immune cell profiling

**Fig. 4:**
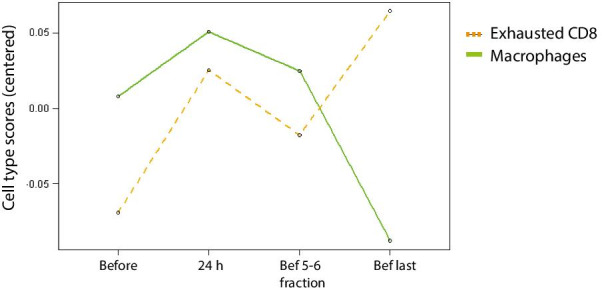
Immune cell profiling analysis indicating cell type abundance measurements versus time points. Raw cell type measurements are calculated as the log2 expression of each cell type’s marker genes

Cell type profiling analysis identified two main cell populations, macrophages and exhausted CD8 + T cells (T cells which adopt a functionally attenuated state due to prolonged antigen stimulation, characteristic of chronic infections and cancer). The average cell type score was compared at the different time points and first and last time points (Fig. 4). The results showed that macrophages were found to be relatively higher before the start of the treatment compared to the last time point but the opposite was found for the exhausted CD8 T cells (Fig. 4).

### Validation of nCounter analysis by RT-qPCR

**Fig. 5:**
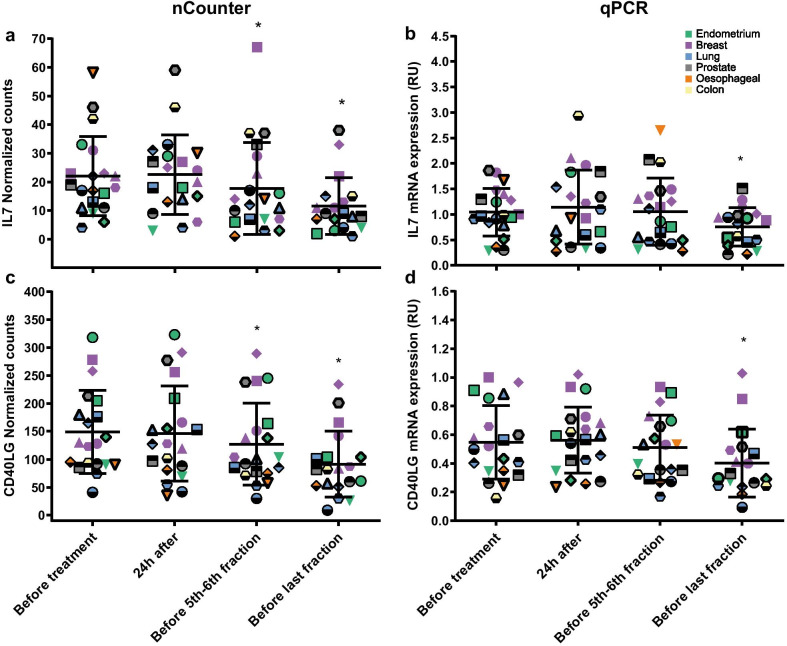
mRNA expression levels of IL7 and CD40LG in blood from radiotherapy patients analysed by nCounter analysis (**a**, **c**) and RT-qPCR (**b**, **d**). Blood samples from 20 patients, comprising those with endometrial, breast, lung, prostate, oesophageal and colon cancer, were analysed. Blood was collected at four time points: before the start of the treatment, at 24 h after the first fraction, before 5th–6th fraction and before the last fraction. Individual data points are shown for all patients, together with the mean ± SD (each patient is represented with a different symbol). Each cancer group was color coded. Statistical analyses were performed in log-transformed data. *Significantly different from the control (before treatment) (paired *t* test, *P* ≤ 0.05). RU, relative units

IL7 and CD40LG were selected to validate the nCounter analysis (Fig. 5). The expression profiles of these genes were confirmed by RT-qPCR with a significant down-regulation at the last time point (last fraction of the radiotherapy treatment).

## Discussion

Our group has previously reported long-term modification of transcriptional expression in genes associated with inflammatory processes in head and neck and endometrial cancer patients undergoing radiotherapy [13].The aim of this study was to report, for the first time in a wide range of cancer types (breast, lung, prostate, endometrium and gastro-intestinal), details of a panel of inflammation-associated genes identified in radiotherapy patients as being significantly associated with ionising radiation exposure which could be further investigated as potential biomarkers of short- and long-term post-exposure. Radiation responsive genes, both up and down regulated, were identified using a Human Inflammation V2 panel from nCounter Analysis system (NanoString Technologies^®^) and assessed for the significance of their individual and combined responses in terms of time since exposure and type of cancer. The radiation doses received varied on a patient by patient basis (full data in Moquet et al. [19]) (Table 1), however, for the purposes of this initial analysis, as each time point pre- and post-exposure was at a different stage of the treatment, time post-exposure can be taken as an analogue for dose. Thus, the results of this work reveal gene sets and pathways which show significance in terms of radiation responsiveness for different groups of patients irrespective of the type of cancer. Both statistical approaches performed in this study identified a common radiation responsive gene, MYC. MYC is a proto-oncogene involved in cell cycle, cell proliferation, apoptosis [24], regulation of innate and adaptive host tumour immune responses [25] and it has been previously described as a radiation responsive gene in different cohorts (head and neck and endometrial cancer patients) [13]. Only MYC and BCL6 were shown to be upregulated in our differential gene expression analysis performed with nSolver advanced analysis software, whereas the MANOVA analysis identified MYC, CD40LG, CCL4, IL7, TCF4, CCR7 and FASLG as significantly differentially expressed. From those genes, CD40LG and IL-7 were observed to be significantly downregulated (before 5th–6th and last RT fraction) using nCounter analysis which was further confirmed by RT-qPCR. However, in RT-qPCR the significance of both CD40LG and IL-7 was observed only before last fraction among all the tumour types. CD40LG is transiently expressed on T cells as a result of inflammatory response and known to activate CD40 [26]. CD40, a member of TNF family is known to be expressed by DC, myeloid cells and B cells and its activation leads to priming of cytotoxic T cells [27]. Recently, IL-7 has been shown to be produced by radioresistant haematopoietic cells in mice [28]. IL-7 regulates homeostasis of lymphocytes, survival and maintenance of T cells [29]. These results suggest that CD40LG and IL-7 have potential as immune-inflammatory radiation exposure biomarkers to correlate dose fraction against volumes irradiated.

In parallel to the identification of the potential biomarkers of radiation exposure, pathway analyses were performed to trace the inflammatory response to radiation. These analyses revealed that there is a modulation of inflammation associated pathways after recurring exposure to radiation during the course of the radiotherapy treatment. It is known that IR can induce inflammation by inducing cytokine secretion and through bystander signals [30–32]. GSA analysis identified as of particular interest two upregulated pathways with a high score at the last time point (before the last fraction), interleukin-18 and class I MHC mediated antigen processing and presentation. Interleukin-18 is involved in activation and differentiation of various T cell populations [33] and its increase has been linked to radiation injury [34]. MHC class I peptides are antigens originated intracellular and delivered to the cell surface to be recognized by CD8 + T cells and an increase in this cell surface peptide presentation has been described after gamma irradiation exposure [35].

Cell markers were present in the nCounter panel for macrophages and exhausted CD8 + T cells. These markers revealed changes in the levels of these cell types during the radiotherapy treatment. Continuous radiation exposure seems to promote a decrease in macrophages at the end of the treatment. Macrophage irradiation has demonstrated to modulate their phenotype towards a pro-inflammatory state promoting cancer angiogenesis and cancer cell-invasion [36]. Macrophage activation and recruitment at site of injury has been proposed as an indirect effect of IR which results from cellular damage signals to clear radiation-induced apoptotic cells [30]. This macrophage recruitment is in line with the slight decrease of macrophages in the early time point of the radiation therapy (24 h after first fraction and before 5th–6th fraction). However, it is not clear why the macrophages decrease after long periods of repetitive exposures in the present study.

T cell exhaustion is an attenuated state of cell-response resulting from repeated or prolonged antigenic exposure under suboptimal conditions [37]. So, it is not surprising to see this cell group increased after the continuous exposure to IR at the end of the radiotherapy treatment.

## Conclusion

In summary, we identified genes associated with inflammatory pathways to be responsive to radiation exposure in blood in vivo. The pathway and cell marker analyses confirmed the activation of an inflammatory response after radiation exposure in vivo. Moreover, these results are encouraging and will be used as part of further research to understand individual radiation responses and explore the links between inflammatory and immune responses in the context of different dose fractionation schedules and volumes irradiated in various cancer types. Ultimately it is hoped these data will help further to develop personalized use of radiation in medicine.

## Data Availability

The datasets used and/or analysed during the current study are available from the corresponding author on reasonable request.

## Abbreviations

ARG1: Arginase 1
BCL2L1: Bcl-2-like protein 1
CCL4: C–C Motif chemokine ligand 4
CCR7: C–C chemokine receptor type 7
CD40LG: CD40 ligand
DE: Differential expression
FASLG: Fas ligand
FDR: False discovery rate
FDXR: Ferredoxin reductase
GSA: Gene set analysis
HPRT1: Hypoxanthine–guanine phosphoribosyltransferase 1
IL7: Interleukin 7
IMRT: Intensity modulated radiotherapy
IR: Ionizing radiation
LINAC: Linear accelerator
MYC: MYC proto-oncogene
RT-qPCR: Quantitative real-time polymerase chain reaction
RT: Radiotherapy
TCF4: Transcription Factor 4
